# Pathobiology of *Salmonella*, Intestinal Microbiota, and the Host Innate Immune Response

**DOI:** 10.3389/fimmu.2014.00252

**Published:** 2014-05-26

**Authors:** Renato Lima Santos

**Affiliations:** ^1^Departamento de Clínica e Cirurgia Veterinárias, Escola de Veterinária, Universidade Federal de Minas Gerais, Belo Horizonte, Brazil

**Keywords:** *Salmonella*, innate immunity, intestinal microbiota, inflammation, enteritis, symbiosis

## Abstract

*Salmonella* is a relevant pathogen under a clinical and public health perspective. Therefore, there has been a significant scientific effort to learn about pathogenic determinants of this pathogen. The clinical relevance of the disease, associated with the molecular tools available to study *Salmonella* as well as suitable animal models for salmonellosis, have provided optimal conditions to drive the scientific community to generate a large expansion of our knowledge about the pathogenesis of *Salmonella*-induced enterocolitis that took place during the past two decades. This research effort has also generated a wealth of information on the host immune mechanisms that complements gaps in the fundamental research in this area. This review focus on how the interaction between *Salmonella*, the microbiota and intestinal innate immunity leads to disease manifestation. As a highly successful enteropathogen, *Salmonella* actively elicits a robust acute intestinal inflammatory response from the host, which could theoretically lead to the pathogen demise. However, *Salmonella* has evolved redundant molecular machineries that renders this pathogen highly adapted to the inflamed intestinal environment, in which *Salmonella* is capable of outcompete resident commensal organisms. The adaptation of *Salmonella* to the inflamed intestinal lumen associated with the massive inflammatory response that leads to diarrhea, generate perfect conditions for transmission of the pathogen. These conditions illustrate the complexity of the co-evolution and ecology of the pathogen, commensals, and the host.

## Introduction

*Salmonella* infection or the disease associated with it, salmonellosis, is most often characterized by enteritis. However, host restricted serotypes tend to induce higher levels of bacteremia, while some human restricted serotypes cause a systemic disease with mild enteric symptoms. All infections in warm blooded animal species and humans are due to one single *Salmonella* species, namely *Salmonella enterica* subsp. *enterica*, which includes more than 2,400 serotypes (1). Currently, there is an effort to reclassify *S. enterica* according to genotypes (based on multilocus sequence typing – MSLT) rather that serotypes. MSLT may be more accurate for predicting pathogenicity and host preferences (2). Although human restricted serotypes (i.e., Typhi and Paratyphi) cause a systemic disease named typhoid fever, several other serotypes, so-called “non-typhoidal *Salmonella*” (NTS) are capable of infecting human patients causing primarily an enteric disease characterized by enteritis and diarrhea. Most of the studies on *Salmonella* enteropathogenesis have been performed with serotype Typhimurium, therefore, unless stated otherwise, this review refers to *Salmonella typhimurium*.

Our understanding of the pathogenic mechanisms of NTS has markedly advanced over the past 20 years. Two important steps were crucial for achieving such advancement: (i) genetic manipulation of the pathogen that allowed researchers to dissect several of the *Salmonella* virulence factors, and (ii) development and characterization of suitable experimental models. Thus, the most significant molecular mechanisms employed by *Salmonella* for invasion and intracellular survival in host cells have been deciphered. *Salmonella* actively invades intestinal epithelial cells. The invasion process requires several effector proteins that are translocated through the *Salmonella* pathogenicity island-1 (SPI-1)-encoded type III secretion system (TTSS) (3, 4). *Salmonella* is also capable of surviving intracellularly in phagocytic and non-phagocytic cells. Intracellular survival requires a second TTSS that is encoded by the *Salmonella* pathogenicity island 2 [SPI-2; (4, 5)].

In parallel to the progress in the field of molecular microbiology, experimental models, including epithelial, phagocytic, and other cell lines (6), as well as the development of animal models were instrumental for advancing in our knowledge on *Salmonella* enteropathogenesis (7, 8). Importantly, there are marked differences on how mammalian hosts respond to *Salmonella* (7). The mouse has been extensively used as a model for experimental infections. Importantly, marked differences in natural resistance has been demonstrated among mouse strains, which is associated with the resistant (e.g., strain 129sv) or susceptible (e.g., strains C57BL6/J and BALB/c) allele of the *Slc11a1* (formerly known as *Nramp1*) gene (9). However, inoculation of mice with *S. typhimurium* results in a systemic infection that is not associated with diarrhea (7), but resembles typhoid fever caused by *S. typhimurium* in human patients (10). Therefore, aside of a few experimental reports with non-human primates (11, 12), bovine experimental infections became very relevant in this context (13) since cattle respond to NTS infection by developing an enteric disease that is clinically similar to human NTS infections (13, 14). Calves can be either orally infected (15) or subjected to surgical ligation of ileal loops that allow for a more precise assessment of early host responses (14). However, experimental studies performed in the 1980s have demonstrated that the absence of the intestinal microbiota has a profound impact on the outcome of infection in the mouse, rendering mice much more susceptible to infection (16). Furthermore, very early experimental studies have demonstrated that mice treated with streptomycin had an increased susceptibility to *Salmonella* (17), which allowed the development of a mouse model of *Salmonella*-induced typhlocolitis based on disruption of the intestinal microbiota by pre-treating the mice with streptomycin prior to challenge with *S. typhimurium* (18). This new model opened the opportunity to largely expand animal experimentation on *Salmonella*-induced intestinal inflammation, but it also clearly demonstrated the profound impact that the intestinal microbiota may have on the pattern of host response and outcome of infection.

The goal of this review is to discuss the advances in our knowledge on the innate intestinal immunity under the light shed by studies on the interaction between *Salmonella*, the intestinal microbiota, and the host.

## Interdependence of the Intestinal Microbiota and the Immune System

During the past few years, it has become increasingly clear that the intestinal microbiota plays a major role modulating intestinal mucosal immunity [reviewed by Ref. (19)]. Mammals coevolved with a complex population of commensal microorganisms that establish a mutually beneficial relationship to an extent that mammalians host more than 10^14^ microorganisms in the intestine (19). The significance of the microbiota for the development of the immune system is illustrated by the several immune defects that are observed in germ free mice, including decreased gut-associated lymphoid tissue, smaller mesenteric lymph nodes, and decreased antibody production, among other structural and functional deficiencies (19). It has been demonstrated that the host specific microbiota is required for full development of the mucosal immunity in the mouse (20). The Th-17 subset of T-cells is required for homeostasis and mucosal integrity, whereas the development of this cell population in the intestine requires the establishment of the microbiota, since germ free mice fail to develop Th-17 in the intestine (21). In a healthy individual, the microbiota prevents translocation of pathogenic microorganisms to the mesenteric lymph node thus preventing an undesirable immune response (22). Disruption of the microbiota (known as dysbiosis) due to antibiotic treatment favors translocation of even a non-invasive mutant *S. typhimurium* strain by phagocytes to the mesenteric lymph node (22).

In the past few years, a large number of relevant scientific reports have clearly established how the pathogen-associated molecular patterns (PAMPs) are recognized by their hosts (ranging from insects to mammalians) through pathogen recognition receptors (PRRs). However, a more recent wave of experimental evidences support the notion that molecules derived from the commensal microbiota are constantly sensed by host PRRs, which is a key step in establishing homeostasis [reviewed by Ref. (23)]. MyD88, a key adaptor protein for most TLRs (toll-like receptors), has been shown to play an important role in this context, since mice lacking MyD88 have a 100-fold increase in the number of bacteria associated with the intestinal mucosa (24). Therefore, considering that commensal microbiota is also sensed by PRRs, the term MAMPs, which stands for microbe-associated molecular patterns, has been proposed (25). Divergence between a PRR-mediated inflammatory response and PRR-mediated immune modulation and homeostasis is dependent on the concurrent presence of additional signals such as stimulation of cytosolic receptors by MAMPs (26). Importantly, in addition to sensing MAMPs, some of the cytosolic PRRs [i.e., Nod-like receptors (NLRs)] are capable of sensing signals associated with cell stress and damage, such as potassium influx, reactive oxygen species, membrane damage, etc. These signals are named danger-associated molecular patterns (DAMPs). Therefore, concomitant stimulation of extracellular PRRs and cytosolic PRRs by MAMPs or DAMPs allows the innate immune system to differentiate between stimuli from the commensal microbiota leading to homeostasis or pathogen triggered responses that lead to inflammation [reviewed by Ref. (27)].

While the establishment of the intestinal microbiota is a key event for immune maturation, conversely, immune cells in the intestine play an active role in shaping the composition of the microbiota, leading to homeostasis [reviewed by Ref. (28)]. For instance, the absence of CD4^+^ T_reg_ cells results in an unregulated T-cell response against antigens from the microbiota, which causes intestinal inflammation (29). Mucosal antibodies, i.e., secretory IgA, also play a central role in shaping the microbiota. Impaired production of high affinity secretory IgA in the intestinal mucosa results in dysbiosis (30). Another very important component of this interaction between the host and microbiota are the intestinal epithelial cells (i.e., enterocytes, goblet cells, and Paneth cells). In addition to a physical barrier, structured by tight junctions between these cells that completely separate the apical from the basolateral compartment, the epithelium generates important factors that modulates expansion and composition of the microbiota. Goblet cells produce large amount of mucous that is a key element in homeostasis, while other cell types, particularly Paneth cells, generate antimicrobial peptides (31).

Interestingly, the influence of the microbiota is not restricted to the intestinal mucosal immunity, but it also impacts systemic immune sites. Antibiotic-induced dysbiosis results in impaired immune response against the influenza virus, while under these circumstances immunity is restored by rectal administration of PPR ligands, indicating that exposure of the intestinal mucosa to MAMPs is critical to modulating immunity (32). Indeed, there are experimental evidences of translocation of MAMPs from the intestine to systemic sites, where it modulates immune maturation, which indicates that the immune modulator role of the intestinal microbiota is not restricted to local tissues, influencing other distant immune organs (33). Interaction of the microbiota with the immune system is extremely complex, to the point that the microbiota may either favor or prevent the development of autoimmune disorders (34) as well as cancer development (35). Furthermore, the microbiota influences numerous other pleiotropic effects, both on pathologic events such as asthma, arthritis, inflammatory bowel diseases, obesity, and cardiovascular disease, as well as on physiological functions including organ morphogenesis, intestinal vascularization, tissue regeneration, bone homeostasis, metabolism, and behavior (36).

## *Salmonella* Interaction with the Intestinal Microbiota

**Figure 1:**
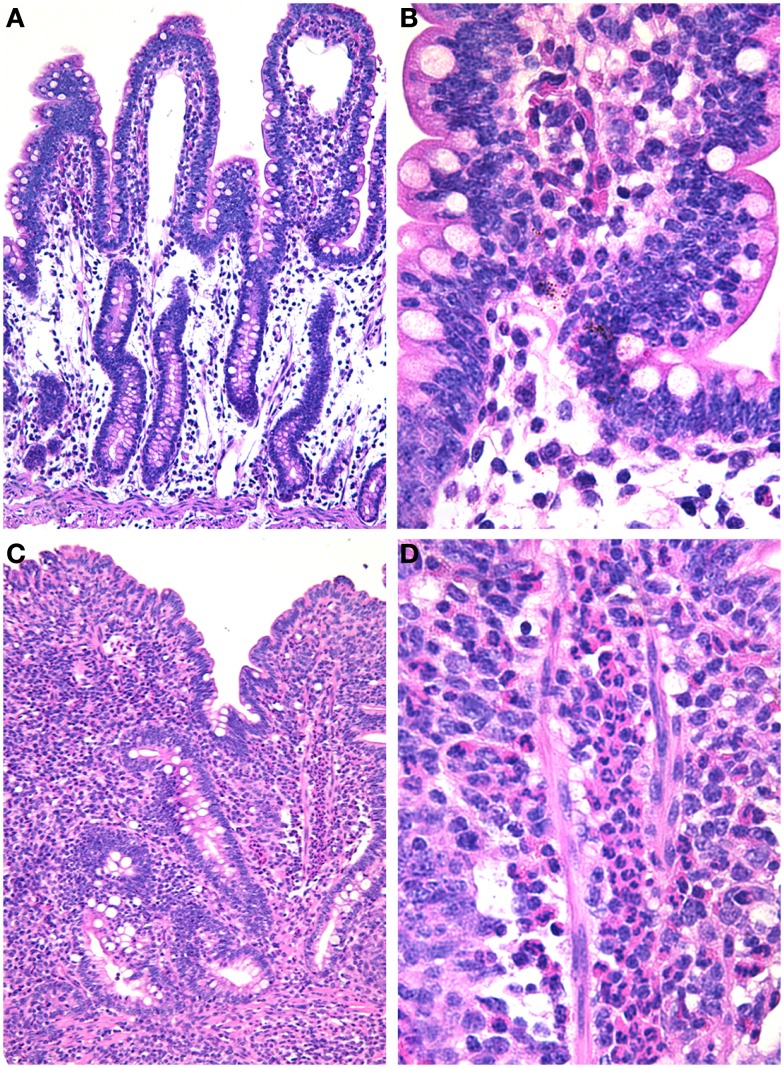
***Salmonella*-induced enteritis in experimentally infected ligated ileal loops in calves**. **(A)** Uninfected loop with no inflammatory reaction; 10× objective. **(B)** Higher magnification of uninfected loop; 40× objective. **(C)** *Salmonella*-infected loop with a severe and diffuse inflammatory infiltrate and blunting of the villi; 10× objective. **(D)** Higher magnification showing a diffuse and severe infiltration of neutrophils; 40× objective. Hematoxylin and eosin.

**Figure 2:**
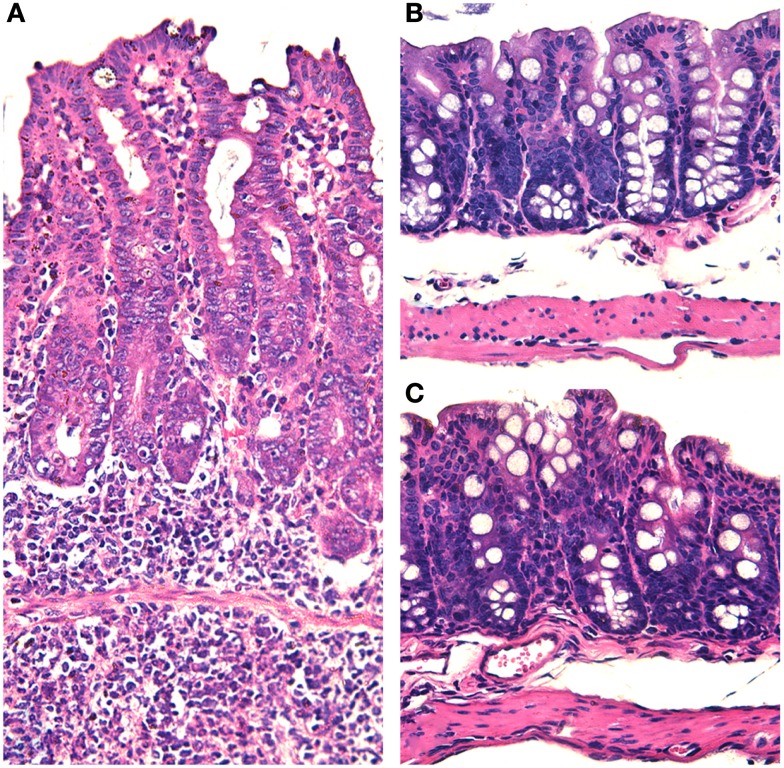
***Salmonella*-induced typhlitis in mice with dysbiotic microbiota due to streptomycin treatment**. **(A)** Marked thickening of the cecal wall with edema and increased cellularity due to a diffuse infiltration of inflammatory cells in a mouse with dysbiosis (pre-treated with streptomycin) and intragastrically infected with *Salmonella enterica* serotype Typhimurium. **(B)** Section of the cecum from a mouse intragastrically infected with *Salmonella enterica* serotype Typhimurium in the presence of a normal microbiota with no histopathological changes. **(C)** Section of the cecum from a healthy non-infected mouse. Note that all micrographs have the same magnification 20× objective. Hematoxylin and eosin.

As previously mentioned, earlier studies have clearly demonstrated that disruption of the intestinal microbiota by treating mice with streptomycin results in increased susceptibility to *Salmonella* infection (17). Furthermore, the intestinal microbiota has a protective effect against *Salmonella* infection in the mouse (16). These studies prompted Barthel et al. (18) to develop a very useful experimental model based on treatment of mice with streptomycin followed by challenge with *S. typhimurium*. This model has been extensively utilized by the entire field, since experimental infections were previously largely restricted to more expensive and labor intensive animal models such as oral infections in calves (15) or the bovine ligated ileal loops (14). Further studies demonstrated that *Salmonella* elicits an inflammatory response in streptomycin-treated mice that is pretty similar to that observed in *Salmonella*-infected germ free mice (37). While *S. typhimurium* infection in cattle triggers an acute inflammatory response that is characterized by massive infiltration of neutrophils (Figure 1) associated with variable degrees of necrosis, hemorrhage, erosion, and fibrinous pseudomembrane formation over the intestinal mucosa, particularly at the ileal Peyer’s patches (14, 15), the same pathogen in the mouse does not elicit significant neutrophilic infiltration in the intestinal mucosa (7). Mice respond to *S. typhimurium* infection with a mild histiocytic infiltration, but in contrast they develop a marked systemic infection that is associated with lesions in the liver and spleen in the absence of diarrhea. Therefore, the development of the streptomycin-treated mouse model largely broadened the possibilities for *in vivo* experimental study of salmonellosis, allowing a marked worldwide expansion of animal experiments among several groups as well as genetic manipulation not just of the pathogen, but also of the host. Pretreatment with streptomycin results in a severe acute inflammatory response of the intestinal mucosa in response to *S. typhimurium* infection (Figure 2) (18). Although the original study that described this model demonstrated that streptomycin-treated mice have a much more efficient intestinal colonization with *S. typhimurium* (18), which suggests that the mechanism is likely due to lack of competition with components of the microbiota, this did not prove any direct cause or effect relationship between composition of the microbiota and the intrinsic nature of the innate intestinal immune response. Therefore, this model opened another extremely important area of investigation in this field, i.e., the role of the microbiota in the pathogenesis of NTS-induced enterocolitis.

Clinical treatment of human patients with antibiotics is recognized as a risk factor for subsequent *Salmonella* infection (38), which correlates well with what we have learned from the streptomycin-treated mouse model of *Salmonella* infection (18). However, the interaction of *Salmonella* with the microbiota is complex, and under certain circumstances pathogen and commensal may not necessarily have a mutually excluding relationship. For instance, a recent study demonstrated that carbohydrates metabolized by commensal microorganisms may serve as energy source for *Salmonella*. In that study, *Bacteroides thetaiotaomicron*, which encodes sialidase that is required to release sialic acid from glycoconjugates, but does not have the enzymatic machinery to utilize sialic acid as a carbon source, generates free sialic acid, whereas *S. typhimurium* that lacks sialidase is capable of catabolizing this carbohydrate (39). *S. typhimurium* can also metabolize fucose generated in a similar manner. Therefore, members of the commensal microbiota are capable of releasing carbon sources that themselves cannot utilize, but that can be used as energy source by *Salmonella* (39). This process is thought to play a role in post antibiotic expansion of enteropathogens (39).

Susceptibility to different enteric pathogens is highly variable among different age groups. Interestingly, these differences in susceptibility may at least in part be related to changes in the composition of the intestinal microbiota [reviewed by Ref. (40)]. During early infancy the microbiota is highly dynamic, whereas in adults it is much more stable and composed predominantly by the phylum Bacteroidetes and Firmicutes (approximately 95% of the microbiota), and elderly tend to have a predisposition to mild inflammation in the intestinal mucosa and decrease in the relative abundance of Actinobacteria (essentially *Bifidobacteria*) and Firmicutes (40). Importantly, early and late stages of life, when the intestinal microbiota is less stable, with relatively lower numbers of Bacteroidetes and relatively higher numbers of gamma Proteobacteria, correspond to the period of higher susceptibility to some enteric pathogens (40).

With an elegant experimental approach, Chung et al. (20) demonstrated that germ free mice associated with human intestinal microbiota have increased susceptibility to *Salmonella* when compared to mice that had been associated with a normal mouse microbiota, indicating that under those experimental conditions the mouse microbiota is more protective against *Salmonella* than the human counterpart. This somewhat parallels the manifestation of *Salmonella*-induced intestinal pathology in these two host species, supporting the notion that the human microbiota may favor *Salmonella*-elicited intestinal inflammation, whereas the murine microbiota impairs the ability of *Salmonella* for triggering a host inflammatory reaction (7), which may be due to a lower antagonistic potential of the human microbiota when compared to that of mice.

An increasing number of experimental evidences points toward the notion that *Salmonella* has evolved multiple mechanisms by which it can overgrow members of the microbiota under conditions of an inflamed intestine (41). Several studies have identified *Salmonella* effectors, among other bacterial factors, that play a role in triggering host inflammation in the intestine (42). *Salmonella*-induced enteropathogenesis is strongly associated with the ability of the pathogen to invade epithelial cells and the intestinal mucosa. Therefore, five effector proteins translocated through the (SPI-1)-encoded TTSS, namely SipA, SopA, SopB, SopD, and SopE2, are required for invasion and enteropathogenesis (43). Earlier studies have demonstrated that *Salmonella* has a competitive advantage over the microbiota in the inflamed intestine, whereas such advantage does not take place in the absence of inflammation (44). Quite a few mechanisms by which *Salmonella* takes advantage of intestinal inflammation have emerged recently. Lipocalin-2, a host antimicrobial peptide, is generated in the inflamed intestine in response to IL-17 and IL-22, whose production is triggered by *Salmonella* infection. This peptide prevents iron acquisition by intestinal microorganisms. It binds enterobactin, a siderophore produced by several enteric bacteria. However, *Salmonella* produces salmochelin (in addition to enterobactin), another siderophore that is not bound by lipocalin-2. Thus, under conditions of inflammation and abundance of lipocalin-2, *Salmonella* has a competitive advantage over other intestinal bacteria (45). Iron deprivation in the inflamed intestine induces expression of colicin Ib by *Salmonella*, which is a bacteriocin active against other *Enterobacteriaceae*, providing additional competitive advantage to *Salmonella* against part of the commensal microbiota under inflammatory conditions (46). Among other mechanisms by which *Salmonella* overgrow the commensal microbiota in the inflamed intestinal environment is based on its ability to acquire microelements, including zinc (47). In the inflamed intestine, calprotectin produced by neutrophils inhibits bacterial growth by sequestering zinc. However, *Salmonella* is capable of evading this host protective mechanism by expressing a high affinity zinc transporter named ZnuABC (47).

Another striking example of *Salmonella* adaptation to intestinal inflammation was provided by Winter et al. (48), who demonstrated that the inflamed intestinal environment provides a respiratory electron acceptor for *Salmonella*. Tetrathionate has been used as an enrichment medium for *Salmonella* isolation *in vitro* from samples containing competitive microbes since the 1920s. Reactive oxygen species generated during the inflammatory process triggered by *Salmonella* itself, oxidizes endogenous thiosulfate to generate tetrathionate, which can then be utilized as an anaerobic respiratory electron acceptor by *Salmonella* (48). This mechanism provides competitive advantage for *Salmonella* in the inflamed intestine while members of the microbiota perish due to environmental changes resulting from the massive *Salmonella*-induced inflammatory response. *Salmonella*-induced inflammation is associated with detachment of large numbers of enterocytes from the mucosa (14), Interestingly, ethanolamine derived from phosphatidylethanolamine, the most abundant phospholipid in membranes of detached enterocytes, can be utilized by *Salmonella* under anaerobic conditions using tetrathionate as electron acceptor in the inflamed gut (49). In addition to tetrathionate respiration, the effector protein SopE induces nitrate production by the host, which favors growth of *Salmonella* by allowing anaerobic nitrate respiration (50). Neutrophil-derived elastase, which is abundant in the inflamed intestine, suppresses components of the commensal microbiota, favoring intestinal growth of *Salmonella* (51). Mechanisms of *Salmonella* adaptation to the inflamed intestinal environment have been recently reviewed by Winter and Bäumler (52).

As a component of the innate host immune response, the inflammatory process should be seen as a host mechanism for preventing the spread of infection, which to some extent is completely correct, since in the absence of a neutrophilic response, *Salmonella* tends to spread more efficiently to systemic sites of infection, both in the mouse (53) as well as in cattle (54). These experimental observations parallel clinical disease since serotype Typhi that causes systemic infections does not elicit a significant intestinal neutrophilic response (10). However, as paradoxically as it may first seem, *Salmonella* evolved to take advantage of the host intestinal inflammatory response. Together, the studies discussed above clearly support the notion that *Salmonella*-induced inflammation is part of this pathogen strategy to create a highly favorable environment in the intestinal lumen for its own multiplication. However, *Salmonella* is a facultative intracellular pathogen, and that interaction with host cells is a determinant of the pathogenic capacity of this organism. Earlier studies strongly focused on the interaction of *Salmonella* with different host cell types, both *in vitro* and *in vivo* (6), missing a very important aspect of the big picture, which is the fact that only a fraction of the *Salmonella* population in a given host actually invades the mucosa during the acute phase of infection, while most of the organisms remain in the intestinal lumen (41). Excessive invasion of the intestinal mucosa by a larger fraction of the population of *Salmonella* could not be desirable under the pathogen point of view, since once within the host tissues, *Salmonella* is exposed to several efficient bactericidal mechanisms. This may explain the role of the SptP effector protein that reverses some of the molecular mechanisms used by *Salmonella* to invade intestinal epithelial cells (55).

Summarizing, *Salmonella* uses a *kamikaze* strategy based on a small fraction of its infecting population actively invading and triggering a massive acute inflammatory response. While this acute neutrophilic response may effectively restrict the infection mostly to enteric sites, largely preventing survival of invasive bacteria, and therefore preventing systemic dissemination of the pathogen, it also creates an intraluminal intestinal environment that favors the remaining larger fraction of the pathogen population that stays in the intestinal lumen, being able to multiply and effectively transmit the infection to the next host.

## Manipulation of the Microbiota for Prophylactic and Therapeutic Purposes

A thorough review of prophylactic and therapeutic approaches to modulate the function and/or composition of the microbiota is completely beyond the scope of this article. However, under a clinical point of view, it is relevant to point out some of the advances in this area. Clinical applications of probiotic and prebiotic has been recently reviewed by Vieira et al. (56). Probiotics are defined as live microorganisms which when administered in adequate amounts confer health benefits to the host [FAO/WHO, 2002 FAO/WHO Working Group, Guidelines for the Evaluation of Probiotics in Food (2002). London, ON, Canada]. The notion of probiotic has been developed long time ago with the original observations of Metchnikoff in the beginning of the twentieth century, who identified microorganisms, particularly *Bacillus bulgaricus* (currently named *Lactobacillus bulgaricus*), which has beneficial effects on health and was the foundation of the yogurt industry (57). Probiotics, including different formulations and several different microorganisms in variable combinations, such as *Saccharomyces boulardii, Bifidobacterium* spp., *Streptococcus thermophilus, Lactobacillus* spp., *Escherichia coli* strain Nissle 1917, among several other microorganisms have been extensively used experimentally or therapeutically for treating enteric diseases with predominantly positive outcomes (56). However, particularly in immune compromised patients, the risk of sepsis should be taken in account when electing a probiotic therapeutic protocol (58). Prebiotics are food ingredients that are not digestible by the host and have favorable effects on specific components of the microbiota and intestinal homeostasis, although this concept may be expanded to include other food ingredients that do not completely fit the criteria for a prebiotic, but have similar effects, such as dietary fibers. Therapeutic or prophylactic combinations of probiotics and prebiotics are termed symbiotics (56).

A similar concept is linked to the ancient therapeutic practice of adoptive transfer of commensal microbiota from healthy individuals to patients with enteric diseases, particularly those associated with antibiotic therapy, which may be successful under certain conditions (59).

Specifically considering salmonellosis, there are experimental evidences indicating that probiotics may have a protective effect in mice experimentally challenged with *Salmonella*. Both germ free and conventional mice pre-treated with *Saccharomyces cerevisiae* UFMG 905 had lower levels of *S. typhimurium* dissemination upon experimental infection (60). Similarly, *Lactobacillus acidophilus* has protective effects against *S. enteritidis* infection in the mouse (61). Although it is not clear whether probiotics will ever have useful therapeutic applications in human patients infected with *Salmonella*, these experimental studies are relevant since probiotics and prebiotics have a significant potential for the animal industry, particularly for poultry and pigs. In food producing animal species, probiotics and prebiotics may prevent a high burden of *Salmonella*, thus mitigating the risk of transmission, with the additional significant benefit of decreasing the need and therefore the exposure of food producing animals to antibiotic treatment and growth promoters, which prevent emergence of antibiotic-resistant strains of pathogens. Indeed, several probiotics as well as food additives have been extensively studied under field conditions, but the results are highly variable, and strongly influenced by management, nutrition, environmental conditions, and obviously the levels of *Salmonella* challenge. Therefore, a general recommendation or a well-established protocol for probiotic or prebiotic prevention of *Salmonella* infection in farm animals is still unavailable (62, 63).

## Concluding Remarks and Perspectives

Since the first identification of microorganisms of the genus *Salmonella* in the beginning of the last century, a large body of knowledge has been accumulated regarding microbiological features of the organism, disease manifestation in different host species as well as its epidemiological implications. However, it was only during the last decade of the past century that molecular tools became available for dissecting pathogenic mechanisms of *Salmonella*. These molecular approaches preceded more sophisticated animal models, and therefore the pioneer investigations on *Salmonella* pathogenesis pictured a pathogen highly specialized in invasion and induction of a host response, as if the pathogen was indifferent to the myriad of commensal microorganisms in the intestinal environment. A subsequent wave of well-designed studies began to reveal, at a mechanistic level, some of the interactions between *Salmonella* and the microbiota in the intestine. Currently, it is clear that the complexity of these processes is unimaginable at this point so this is still a broadly open field for scientific investigation. A deeper knowledge of the pathobiology of *Salmonella* in the context of the intestinal environment may certainly open new perspectives for therapeutic approaches as well as for controlling animal and human salmonellosis.

## Conflict of Interest Statement

The author declares that the research was conducted in the absence of any commercial or financial relationships that could be construed as a potential conflict of interest.
